# Discovery and Characterization of VU0542270, the First Selective Inhibitor of Vascular Kir6.1/SUR2B K_ATP_ Channels[Fn fn5]

**DOI:** 10.1124/molpharm.123.000783

**Published:** 2024-03

**Authors:** Kangjun Li, Samantha J. McClenahan, Changho Han, Joseph D. Bungard, Upendra Rathnayake, Olivier Boutaud, Joshua A. Bauer, Emily L. Days, Craig W. Lindsley, Elaine L. Shelton, Jerod S. Denton

**Affiliations:** Departments of Anesthesiology (K.L., S.J.M., J.S.D.), Pharmacology (K.L., C.H., J.D.B., U.R., O.B., C.W.L., J.S.D.), Pediatrics (E.L.S.), and Biochemistry (J.A.B.), Vanderbilt University Medical Center, Nashville, Tennessee and Vanderbilt Institute of Chemical Biology, Vanderbilt University, Nashville, Tennessee (J.A.B., E.L.D., J.S.D.)

## Abstract

Vascular smooth muscle K_ATP_ channels critically regulate blood flow and blood pressure by modulating vascular tone and therefore represent attractive drug targets for treating several cardiovascular disorders. However, the lack of potent inhibitors that can selectively inhibit Kir6.1/SUR2B (vascular K_ATP_) over Kir6.2/SUR1 (pancreatic K_ATP_) has eluded discovery despite decades of intensive research. We therefore screened 47,872 chemically diverse compounds for novel inhibitors of heterologously expressed Kir6.1/SUR2B channels. The most potent inhibitor identified in the screen was an *N*-aryl-*N*’-benzyl urea compound termed VU0542270. VU0542270 inhibits Kir6.1/SUR2B with an IC_50_ of approximately 100 nM but has no apparent activity toward Kir6.2/SUR1 or several other members of the Kir channel family at doses up to 30 µM (>300-fold selectivity). By expressing different combinations of Kir6.1 or Kir6.2 with SUR1, SUR2A, or SUR2B, the VU0542270 binding site was localized to SUR2. Initial structure-activity relationship exploration around VU0542270 revealed basic texture related to structural elements that are required for Kir6.1/SUR2B inhibition. Analysis of the pharmacokinetic properties of VU0542270 showed that it has a short in vivo half-life due to extensive metabolism. In pressure myography experiments on isolated mouse ductus arteriosus vessels, VU0542270 induced ductus arteriosus constriction in a dose-dependent manner similar to that of the nonspecific K_ATP_ channel inhibitor glibenclamide. The discovery of VU0542270 provides conceptual proof that SUR2-specific K_ATP_ channel inhibitors can be developed using a molecular target-based approach and offers hope for developing cardiovascular therapeutics targeting Kir6.1/SUR2B.

**SIGNIFICANCE STATEMENT:**

Small-molecule inhibitors of vascular smooth muscle K_ATP_ channels might represent novel therapeutics for patent ductus arteriosus, migraine headache, and sepsis; however, the lack of selective channel inhibitors has slowed progress in these therapeutic areas. Here, this study describes the discovery and characterization of the first vascular-specific K_ATP_ channel inhibitor, VU0542270.

## Graphical abstract

**Figure:**
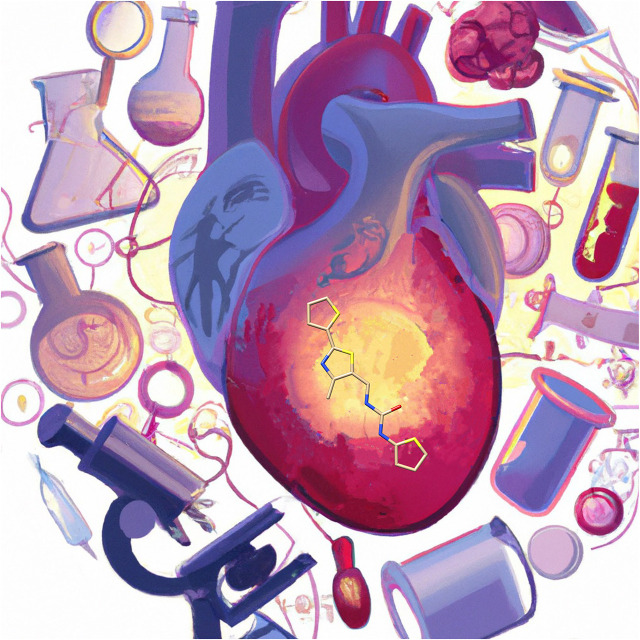

## Introduction

ATP-sensitive potassium channels (K_ATP_) are regulated by intracellular nucleotide concentrations and thus serve to couple metabolic state to membrane excitability in diverse cell types (Nichols, 2006; Davis et al., 2022; McClenaghan and Nichols, 2022). K_ATP_ channels are hetero-octameric complexes composed of four pore-forming inward rectifier potassium (Kir) channel subunits, Kir6.1 or Kir6.2, and four regulatory sulfonylurea receptor (SUR) subunits, SUR1, SUR2A, or SUR2B (Inagaki et al., 1997; Shyng and Nichols, 1997; Aguilar-Bryan and Bryan, 1999; Martin et al., 2017). Kir6.1 and Kir6.2 are encoded by *KCNJ8* and *KCNJ11*, respectively. SUR1 is encoded by *ABCC8*, whereas SUR2A and SUR2B are carboxyl-terminus splice variants of *ABCC9*. Different combinations of Kir and SUR subunits give rise to functionally and pharmacologically distinct K_ATP_ channel subtypes that are expressed in a cell type–specific manner, creating opportunities for developing selective therapeutics targeting different organ systems (Gribble et al., 1997, 1998; Inagaki et al., 1995, 1996; Nichols, 2023).

The best-known example of this is the Kir6.2/SUR1 channel subtype expressed in insulin-secreting *β* cells of the pancreas (Inagaki et al., 1995). In the fasting state, Kir6.2/SUR1 channels are kept open by the low concentration of intracellular ATP relative to ADP. However, the influx of glucose following a meal and ensuing stimulation of ATP production leads to inhibition of Kir6.2/SUR1, membrane potential depolarization, opening of voltage-gated calcium channel and calcium influx, exocytotic release of insulin into the circulation, and lowering of blood glucose levels back toward preprandial levels (Nichols, 2006). Sulfonylurea drugs, such as glibenclamide, that inhibit Kir6.2/SUR1 have been used clinically for decades to treat type 2 diabetes due to their ability to stimulate insulin secretion and lower blood glucose (Kharade et al., 2016; Nichols, 2023).

Kir6.1/SUR2B channels are expressed primarily in vascular smooth muscle cells, where they play critical roles in regulating vascular tone, blood pressure, and blood flow (Quayle et al., 1997; Yamada et al., 1997; Cui et al., 2002; Li et al., 2003; Aziz et al., 2014; McClenaghan and Nichols, 2022). Like pancreatic K_ATP_ channels, vascular K_ATP_ channels also couple metabolic energy status to cell excitability; however, Kir6.1/SUR2B opening and closing lead to vasodilation and vasoconstriction of the vasculature, respectively. Consequently, Kir6.1/SUR2B is an attractive drug target for therapeutics designed to work by modulating vascular tone (Kharade et al., 2016; Nichols, 2023). One potential therapeutic application of vascular-specific K_ATP_ channel inhibitors is the treatment of patent ductus arteriosus (PDA) in newborns (Shelton et al., 2018). The ductus arteriosus (DA) is a fetal artery that diverts blood supply away from the fluid-filled lungs and toward the placental circulation for gas exchange. Following the first breath at birth, an increase in blood oxygen and reduction in prostaglandin levels promotes DA closure, which, in turn, redirects the blood supply to the newly inflated lungs for gas exchange. PDA, resulting from the failure of the DA to contract and close, is one of the most common congenital heart conditions, affecting approximately 1 in 2000 births and up to 10% of all congenital heart diseases (Dice and Bhatia, 2007). Microarray analysis identified *KCNJ8* and *ABCC9* transcripts as being enriched in DA tissues relative to other vessels, suggesting that Kir6.1/SUR2B channels play important roles in regulating DA tone (Shelton et al., 2014; Yarboro et al., 2018). Gain-of-function mutations in *KCNJ8* or *ABCC9* result in Cantu syndrome, a rare genetic disorder characterized by excessive hair growth, distinctive facial features, and an enlarged heart (Harakalova et al., 2012; van Bon et al., 2012; Brownstein et al., 2013; Li et al., 2013; Nichols et al., 2013; Cooper et al., 2014). More than 50% of Cantu patients are born with symptomatic PDA (McClenaghan and Nichols, 2022). Taken together, these observations indicate that K_ATP_ channels are functionally expressed in DA tissues and represent potential drug targets for treating PDA. However, the lack of vascular-specific K_ATP_ channel inhibitors represents a critical barrier to testing this model, prompting us to take a molecular target-based approach to developing novel Kir6.1/SUR2B inhibitors. Here, we report the discovery and characterization of the first potent and selective vascular K_ATP_ channel inhibitor, VU0542270, and demonstrate that it induces vasoconstriction in mouse DA vessels.

## Materials and Methods

### Plasmids and Cell Lines

The pcDNA3.1-SUR2B plasmid was generously provided by Dr. Colin Nichols (Washington University). The pcDNA5/TO-Kir6.1 plasmid was synthesized by GenScript. Stably transfected tetracycline-regulated expression human embryonic kidney-293 (T-Rex-HEK293) cells expressing both plasmids were generated by cotransfecting cells with Lipofectamine LTX followed by antibiotic selection and clonal selection using the thallium flux assay described below. Monoclonal lines expressing robust pinacidil-activated thallium flux were selected for high K_ATP_ channel activity. T-Rex-HEK293 cells expressing Kir6.2/SUR1 were created as described previously (Raphemot et al., 2014). The cell culture media contained Dulbecco’s modified Eagle’s medium (Gibco, 11965-092) supplied with heat-inactivated FBS (bio-techne, Minneapolis, MN S11150H,10%), Blasticidine HCl (Gibco, A11139-03, 10 ug/mL), Penicillin-Streptomycin (Gibco, 15140-122, 2 mM), G418 Sulfate (CORNING, Corning, NY, 30-234-CR, 1 mg/mL), and Hygromycin B (Invitrogen, Carlsbad, CA, 10687-010, 250 ug/mL). For selectivity assays, HEK293T cells were cotransfected with Kir6.x and SURX with Lipofectamine LTX and incubated for 48 hours before thallium flux assays.

### Thallium Flux Assays

Quantitative thallium flux assays of Kir6.1/SUR2B activity were performed essentially as described previously for Kir6.2/SUR1 (Raphemot et al., 2014; Kharade et al., 2019). Stable T-Rex-HEK293-Kir6.1/SUR2B cells or transiently transfected HEK293T cells were plated in polyamine-coated, clear-bottomed, black-walled 384-well plates and were cultured (37°C/5% CO_2_) overnight in Dulbecco’s modified Eagle’s medium (Gibco, Carlsbad, CA, 11995-065) containing 10% FBS (2 mM Penicillin-Streptomycin). The following day, cells were washed with assay buffer (i.e., Hanks’ balanced salt solution/20 mM HEPES) and loaded for 1 hour with thallium sensor dye Brilliant Thallium (Ion Bioscience, San Marcos, TX) at room temperature. Dye-loaded cells were washed with assay buffer and transferred to a Panoptic Kinetic Imaging Plate Reader (Wavefront Bioscience, Franklin, TN). Control and test compound treatments were for 4 minutes prior to adding 0.5 mM chloride-free thallium stimulus buffer (Ion Bioscience) to initiate thallium flux. Live-cell measurements were collected at 1 Hz (482/35 nm excitation and 536/40 nm emission) for 6 minutes. The following control activators and inhibitor were used for assay development and library screening: pinacidil (10 µM; SUR2-specific opener), VU0071063 (30 µM; SUR1-specific opener) (Raphemot et al., 2014), and glibenclamide (10 µM; SUR1/SUR2 inhibitor). Selectivity assays against other Kir channels were performed essentially as described previously (Kharade et al., 2018; McClenahan et al., 2022).

### High-Throughput Screening

Test compounds from the Vanderbilt Institute of Chemical Biology (VICB) Discovery Collection were screened against Kir6.1/SUR2B in singlicate at a nominal concentration of 10 µM. Data were acquired using Panoptic Software, Waveguide (Wavefront Biosciences). Raw fluorescence values were imported for analysis into custom software (VICB, Vanderbilt University). Fluorescence versus time values for each well were normalized to the initial fluorescence value [F/F_0_]. Slopes of the normalized data were calculated for each well between 5 and 12 seconds following thallium stimulation and normalized to the percentage of maximal activator (pinacidil or VU0071063) response for each 384-well plate. Screening hits were defined as compounds that meet both Z-score and Robust Z-score criteria (i.e., 3 standard deviations from the mean and 3 mean absolute deviations of the median) and do not have activity prior to thallium addition (e.g., no fluorescent tags). Hits that retested positive and were negative against nontransfected T-Rex-HEK293 cells and Kir6.2/SUR1 cells were tested in triplicate in nine-point, threefold dilution concentration-response curves (CRCs) ranging between 30 µM and 5 nM. CRC data were plotted in GraphPad Prism (GraphPad Software, San Diego, CA) and fitted with a four-parameter logistic model to determine IC_50_ values for rank-ordering hit potency.

### Whole-Cell Patch Clamp Electrophysiology

T-Rex-HEK293-Kir6.1/SUR2B cells were plated into tissue culture-treated 35-mm polystyrene dishes at a density of 250,000 cells and cultured in a 5% CO_2_ incubator at 37°C. Whole-cell patch-clamp recordings were performed 48 hours after plating. Cells were dissociated on the experiment day and plated on poly-L-lysine–coated coverslips and allowed to recover for at least 1 hour in a 37°C/5% CO_2_ incubator before beginning experiments. Patch electrodes (2 to 3 MΩ) were filled with an intracellular solution containing 130 mM KCl, 2 mM MgCl_2_, 1 mM EGTA, 20 mM HEPES-free acid, and 2 mM Na_2_ATP (Roche Diagnostics, Risch-Rotkreuz, Switzerland), pH 7.3, titrated with KOH, and osmolarity 275 mOsmol/kg of water (adjusted with sucrose). The standard bath solution contained 135 mM NaCl, 5 mM KCl, 2 mM CaCl_2_, 1 mM MgCl_2_, 5 mM glucose, and 10 mM HEPES-free acid, pH 7.4, titrated with NaOH. Serial dilutions of compounds were first made in DMSO and then in bath solution so that the final DMSO concentration was 0.1% v/v across all test doses. Macroscopic currents were recorded under voltage-clamp conditions using an Axopatch 200B Amplifier (Molecular Devices, Sunnyvale, CA). Data were collected at 5 kHz and filtered at 1 kHz. A step protocol was used to generate current-voltage curve relationships. Cells were voltage-clamped at a holding potential of −75 mV and then stepped every 200 milliseconds from −120 mV to 120 mV in 20-mV increments. A ramp protocol was used to generate the dose-response curves. Cells were voltage-clamped at a holding potential of −75 mV, stepped to −120 mV for 200 milliseconds, ramped from −120 mV to 120 mV at a rate of 1.2 mV/msec, and held at 120 mV for 15 milliseconds before being stepped to −120 mV for 100 millisecond. This voltage protocol was repeated every 5 seconds. Pharmacology experiments were terminated by applying the nonselective K_ATP_ channel inhibitor glibenclamide (10 µM) to block the expressed currents and measure residual leak current. Cells exhibiting <90% block by glibenclamide were excluded from the analysis. Data acquisition and analysis were performed using the pClamp 9.2 software suite (Molecular Devices). For the step protocol, mean current amplitude during the last 50 milliseconds of the 150-milliseconds step was used for current-voltage (IV) calculations. For the ramp protocol, mean current amplitude of the cell at 120 milliseconds over 15 milliseconds was used. IC_50_ values were determined by fitting the Hill equation to CRCs using variable-slope nonlinear regression analyses. All the analyses were performed with GraphPad Prism version 5.01 (GraphPad Software).

### Mice

CD-1 mice were maintained at Vanderbilt University Medical Center in accordance with protocols approved by the Institutional Animal Care and Use Committee. Timed matings were performed to produce offspring at specific stages of development. The presence of a vaginal plug was designated as embryonic day 1.

### DA Vessel Myography

Term-gestation (embryonic day 19) fetal mouse DAs were isolated and mounted on glass pipet tips in microvessel perfusion chambers as previously reported (Hooper et al., 2016). Deoxygenated Krebs buffer was warmed to 37°C and circulated throughout the chamber. Vessels were pressurized in a stepwise manner to physiologic neonatal mouse mean arterial pressure (20 mmHg) and then challenged with 50 mM KCl to test reactivity. Vessels that failed to constrict were considered nonviable and excluded from further study. For dose-response studies, vessels were first dilated by circulating Krebs buffer bubbled with 95% nitrogen, 5% CO_2_ to mimic the hypoxic conditions of the womb. Vessels were then exposed to increasing concentrations of glibenclamide (10^−9^M–10^−4^M) or VU0542270-1 (10^−9^M–10^−5^M). Following each dose, lumen diameters were allowed to plateau (approximately 20 minutes) before the next dose was added. Continuous lumen diameter measurements were recorded using a digital image capture system (IonOptix). An N of >6 vessels from at least three different litters was used for each experimental condition. Changes in lumen diameter are represented as the percent change in diameter compared with the baseline lumen diameter of vessels prior to drug exposure. Control experiments were repeated by treating vessels with increasing concentrations of glibenclamide or VU0542270-1 in the presence of 10 µM pinacidil. Values represent mean ± S.D.

### Chemistry

Synthesis of VU0542270 and analogs for structure-activity relationship (SAR) are described in Supplemental Fig. 1.

### Pharmacokinetics of VU0542270 in Rats after Intravenous Administration

All animal housing and experimental procedures were approved by the Vanderbilt University Animal Care and Use committee and followed the guidelines set forth by the *Guide for the Care and Use of Laboratory Animals*. VU0542270 was formulated as a solution in ethanol, PEG400, and saline (1:4:5 v/v, respectively) at a concentration of 1 mg/mL and administered as a single 1-mg/kg IV dose (1 mL/kg) to male Sprague Dawley rats (*n* = 2; between 366–418 g body weights) via injection into a surgically implanted jugular vein catheter. Blood samples were collected serially from a surgically implanted carotid artery catheter in each animal over multiple postadministration time points (0.033, 0.117, 0.25, 0.5, 1, 2, 4, 7, and 24 hours) into chilled K2EDTA anticoagulant-fortified tubes and immediately placed on wet ice. The blood samples were then centrifuged [1700 relative centrifugal force (rcf), 5 minutes, 4°C] to obtain plasma samples, which were stored at −80°C until analysis by liquid chromatography tandem mass spectrometry (LC-MS/MS). The intravenous pharmacokinetic (PK) data were used for the calculation of relevant intravenous pharmacokinetic parameters [plasma clearance (CL_p_), volume of steady state, elimination half-life (*t*_1/2_), and mean residence time]. In vivo pharmacokinetic parameters of VU0542270 were determined from the observed individual animal time-versus-concentration data using noncompartmental analysis via WinNonlin (WNL; v.5.3, Pharsight Corp., Mountain View, CA.).

### Brain: Plasma Distribution Determination

VU0542270 was formulated as a solution in ethanol, PEG400, and saline (1:4:5 v/v, respectively) and administered to male Sprague Dawley rats (*n* = 1) intravenously at 0.2 mg/kg. At 15 minutes postdose, a blood sample was collected terminally into chilled K_2_EDTA anticoagulant-fortified tubes and immediately placed on wet ice. The blood sample was then centrifuged (1700 rcf, 5 minutes, 4°C) to obtain a plasma sample. At the same postadministration time point(s), a whole-brain sample was obtained by rapid dissection, rinsing with PBS, and immediate freezing in individual tissue collection boxes (dry ice). All brain and plasma samples were stored at −80°C until analysis by LC-MS/MS. In vivo brain:plasma distribution partition coefficient of VU0542270 was determined from the observed animal brain versus plasma concentrations.

### Samples Preparation for Bioanalysis

Plasma samples from the in-life phases of the studies were thawed at ambient temperature (benchtop), and then aliquots (20 µL per sample) were transferred to a 96-shallow-well (V-bottom) plate. Matrix-matched quality-control samples and a standard curve of VU0542270 (1 mg/mL DMSO stock solution) were prepared in blank rat plasma (K_2_EDTA treated) or blank brain homogenate via serial dilution and transferred (20 µL each) to the plate along with multiple blank plasma and brain homogenate samples. Acetonitrile (120 µL) containing internal standard (50 nM carbamazepine) was added to each well of the plate to precipitate protein. The plate was then centrifuged (4000 rcf, 5 minutes, ambient temperature), and resulting supernatants (60 µL each) were transferred to a new 96-shallow-well (V-bottom) plate containing an equal volume (60 µL per well) of water (Milli-Q purified). The plate was then sealed in preparation for LC-MS/MS analysis (see below).

Preparation of brain samples was identical to that of plasma samples except for the following modifications. While thawing, brains were weighed (inside their collection boxes using a universal empty collection box tare weight) and then subjected to mechanical homogenization (Mini-BeadBeater, BioSpec Products, Inc., Bartlesville, OK) in the presence of zirconia/silica beads (1.0 mm) and extraction buffer (isopropanol:water, 7:3, v/v; 3 mL per sample, corrected for postquantitation). Homogenized brain samples were then centrifuged (4000 rcf, 5 minutes, ambient temperature), and the resulting supernatants were diluted in three volumes of plasma (dilution factor of 4). An aliquot (20 µL) was transferred to a 96-shallow-well (V-bottom) plate.

### Binding of VU0542270 in Plasma from Mouse, Rat, And Human

Determination of VU0542270 fraction unbound (f_u_) in plasma from mouse, rat, and human was conducted in vitro via equilibrium dialysis using high-throughput dialysis (HTD) membrane plates. Dialysis membranes (four paired strips per HTD assay) were hydrated as described by the manufacturer and inserted into the HTD plate, which was assembled and prepared for sample addition by the dispensing of blank buffer [Delbecco’s phosphate buffered saline (DPBS), 100 µL/well] into the “top half” of each membrane-split well. VU0542270 was diluted into plasma from each species (5 µM final concentration), which was aliquoted in triplicate to the “bottom half” of the prepared HTD plate wells. The HTD plate was sealed and incubated for 6 hours at 37°C. Following incubation, each well (both top and bottom halves) was transferred (20 µL) to the corresponding well of a 96-shallow-well (V-bottom) plate. The daughter plates were then matrix matched (buffer side wells received equal volume of plasma, and plasma side wells received equal volume of buffer), and extraction solution (120 µL; acetonitrile containing 50 nM carbamazepine as IS) was added to all wells of both daughter plates to precipitate protein and extract test article. The plates were then sealed and centrifuged (3500 rcf) for 10 minutes at ambient temperature. Supernatant (60 µL) from each well of the daughter plates was then transferred to the corresponding wells of new daughter plates (96-shallow-well, V bottom) containing water (Milli-Q, 60 µL/well), and the plates were sealed in preparation for LC-MS/MS analysis (see below).

f_u_ was calculated as (analyte to IS mass spectrometry (MS) peak area ratio from Trans-buffer side)/(analyte to IS MS peak area ratio from Cis-plasma side). Mean values for each species were calculated from three replicates.

### Binding of VU0542270 in Brain Homogenate from Mouse and Rat

Determination of VU0542270 f_u_ in plasma from mouse and rat was conducted using the same methodology and procedure as described for plasma protein binding assay with the following modifications: 1) a final compound concentration of 1 μM was used, and 2) naïve rat brains were homogenized in DPBS (1:3 composition of brain:DPBS, w/w) using a Mini-Bead Beater machine to obtain brain homogenate.

The diluted fraction unbound (fu2) in brain was calculated as (analyte to IS MS peak area ratio from Trans-buffer side)/(analyte to IS MS peak area ratio from Cis-brain homogenate side). Undiluted fraction unbound for the brain was calculated using the following equation:

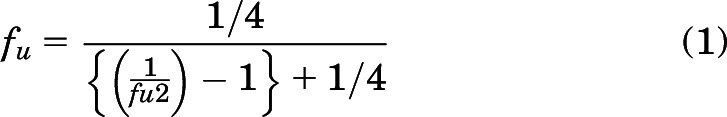


Mean values for each species were calculated from three replicates.

### Intrinsic Clearance of VU0542270 in Rat, Mouse, and Human Liver Microsomes

The in vitro intrinsic clearance (*CL_int_*) of VU0542270 was investigated in commercially obtained hepatic microsomes from rat, mouse, and human donors using the substrate depletion (i.e., loss-of-parent versus time, or *t*_1/2_ method) approach with analyte detection via LC-MS/MS. For each species, mean per cent parent remaining values at each time point were calculated from replicates raw data (analyte:IS peak area ratios) and used to determine in vitro* t*_1/2_ and *CL_int_*.

Experiments were carried out using a robot-assisted liquid handling system (TECAN model Evo 200). VU0542270 was incubated (1 µM final concentration) in buffer (100 mM potassium phosphate, pH 7.4, with 3 mM MgCl_2_) containing hepatic microsomes (0.5 mg/mL final concentration) from multiple species, discretely, at 37°C under constant orbital shaking. After 5 minutes (preincubation), reactions were initiated by the addition of NADPH (1 mM final concentration). At selected time intervals (0, 3, 7, 15, 25, and 45 minutes) post addition of NADPH, aliquots (50 µL) were taken and placed into a 96-shallow-well plate containing ice-cold acetonitrile (150 µL) with carbamazepine (IS, 50 nM). The plates were then centrifuged (3000 rcf at 4°C) for 10 minutes. The supernatants were transferred to a new 96-shallow-well daughter plate and diluted (1:1 v/v) with water (Milli-Q filtered). The plates were then sealed in preparation for LC-MS/MS analysis (see below).

Raw LC-MS/MS peak area data generated from the assay samples were used to construct natural log-transformed per cent parent remaining versus time plots. In vitro VU0542270 *t*_1/2_ values were obtained using the following equation:

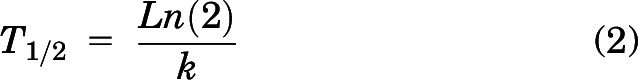
where *k* is the slope from linear regression analysis of the natural log-transformed data (using means from all replicates at each time point). Resulting *t*_1/2_ values were then used to calculate hepatic *CL_int_* values according to the following equation and with the use of species-specific scale-up factors for liver weight (grams) per total body weight (kg):

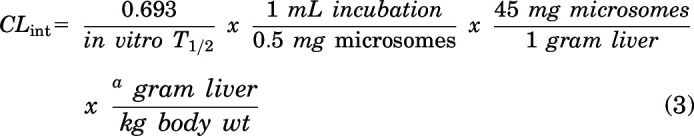


^a^Scale-up factors used are 45 (rat), 87.5 (mouse), and 20 (human) [scaling factors were derived from Lin et al. (1996)].

Predicted hepatic clearance (*CL_hep_*) was calculated using the following equation:

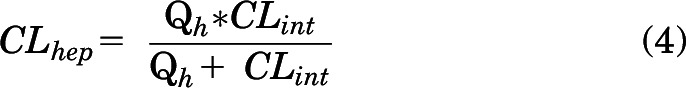


Q_h_ represents hepatic blood flow (ml/min per kg): 21 for human, 70 for rat, and 90 for mouse.

### LC-MS/MS Analysis

Prepared samples were injected (10 µL each) onto an AB Sciex triple quad-4500 mass spectrometer system with an Agilent 1290 Infinity Binary Pump and multisampler. Analytes were separated on a reverse phase column Phenomenex Kinetex C18 (50 × 2.1 mm, 5 µm) that was thermostated at 40°C. High-pressure liquid chromatography mobile phase A was 0.5% formic acid in water (pH unadjusted); mobile phase B was 0.5% formic acid in acetonitrile (pH unadjusted). A 5% B gradient was held for 0.2 minutes and was linearly increased to 90% B over 0.8 minutes, with an isocratic hold for 0.5 minutes, before transitioning to 10% B over 0.05 minutes. The column was re-equilibrated (1 minute) before the next sample injection. The total run time was 2.55 minutes, and the high-pressure liquid chromatography flow rate was 0.5 ml/min. The source temperature was set at 500°C, and mass spectral analyses were performed using a Turbo-Ion spray source in positive ionization mode (5.0-kV spray voltage) and using multiple-reaction monitoring of transitions specific for both analyte (m/z 335.7–236.7 at 35 eV) and internal standard (m/z 237.0–193.9 at 25 eV). Quantitation of VU0542270 was performed via AB Sciex Analyst software using the raw analyte:IS peak area ratios. The typical detection range for VU0542270 was 0.5 ng/mL to ≥5000 ng/mL, utilizing a quadratic equation regression with 1/x^2^ weighting.

Correction for dilution of all brain samples (in extraction buffer and subsequently in blank plasma as previously described) was performed postquantitation. The corrections for dilution in extraction buffer employed correction factors specific to brain weight (2.63×) and to dilution of brain extract in plasma (4×).

## Results

### Kir6.1/SUR2B Thallium Flux Assay

**Fig. 1.**
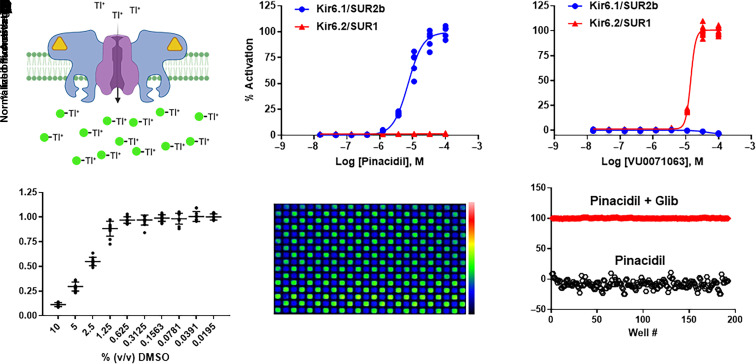
High-throughput screening assay development for Kir6.1/SUR2B. (A) Cartoon depiction of the thallium flux assay used for HTS. Kir6.1 (purple)/SUR2B (blue) channels expressed in HEK-293 cells are opened with pinacidil (yellow triangle) before adding extracellular thallium (Tl^+^) to induce flux through the pore and detection with Thallos Brilliant dye (green circles). (B) CRC data showing activation of Kir6.1/SUR2B, but not Kir6.2/SUR1, with pinacidil (*n* = 9 at each dose). (C) CRC data showing opening of Kir6.2/SUR1, but not Kir6.1/SUR2B, with VU0071063. (D) Sensitivity of thallium assay to increasing doses of DMSO. Individual data points with means ± S.D. (*n* = 9 at each dose) are shown. (E) Pseudo-colored fluorescence image of 384-well plate containing HEK-293-Kir6.1/SUR2B cells treated with 10 µM pinacidil (green wells) or 10 µM pinacidil + 10 µM glibenclamide (blue wells) approximately 30 seconds after thallium addition. (F) Scatter plot of fluorescence values measured from the plate in E1 showing clear separation of the twowell populations (i.e., pinacidil versus pinacidil plus glibenclamide). 0% inhibition is defined as cell treated with 10 µM pinacidil, and 100% inhibition is defined as cell treated with 10 µM pinacidil + 10 µM glibenclamide. The calculated Z’ for this plate was 0.64.

The thallium flux assay measures the inward movement of thallium through the Kir6.1/SUR2B channel pore after opening with pinacidil (Fig. 1A). We confirmed the functional expression of Kir6.1/SUR2B in T-Rex-HEK293 cells by comparing their pharmacological responses to the SUR2-specific opener, pinacidil, and SUR1-specific opener, VU0071063. T-Rex-HEK293-Kir6.2/SUR1 cells were used as a comparison. As expected, pinacidil dose-dependently stimulated Kir6.1/SUR2B-medicated thallium flux with an EC_50_ of 7.3 ± 2.4 µM (*n* = 5 wells/dose) but had no effect on Kir6.2/SUR1 (Fig. 1B). In contrast, VU0071063 opened Kir6.2/SUR1 (EC_50_ = 15.3 ± 0.7 µM; *n* = 5 wells/dose) but not Kir6.1/SUR2B (Fig. 1C). Thus, heterologous expression of Kir6.1/SUR2B in HEK293 cells recapitulates the pharmacological properties of vascular K_ATP_.

Kir6.1/SUR2B-mediated thallium flux was tolerant to DMSO at doses up to 0.625% v/v (Fig. 1D), indicating that DMSO has no confounding effects at a screening concentration of 0.1% v/v. Assay uniformity was evaluated in fluorescent checkerboard assays where every other well of a 384-well plate was treated with 10 µM pinacidil or 10 µM pinacidil plus 10 µM glibenclamide (Fig. 1E) before stimulating thallium flux. A plot of thallium-induced fluorescence from activated (pinacidil) and inhibited (pinacidil + glibenclamide) wells is shown in Fig. 1F, demonstrating a clear separation of the two cell populations. The average Z’ calculated from 150 plates over 10 days was 0.57, showing that the assay is robust and reproducible.

### Discovery of VU0542270

**Fig. 2.**
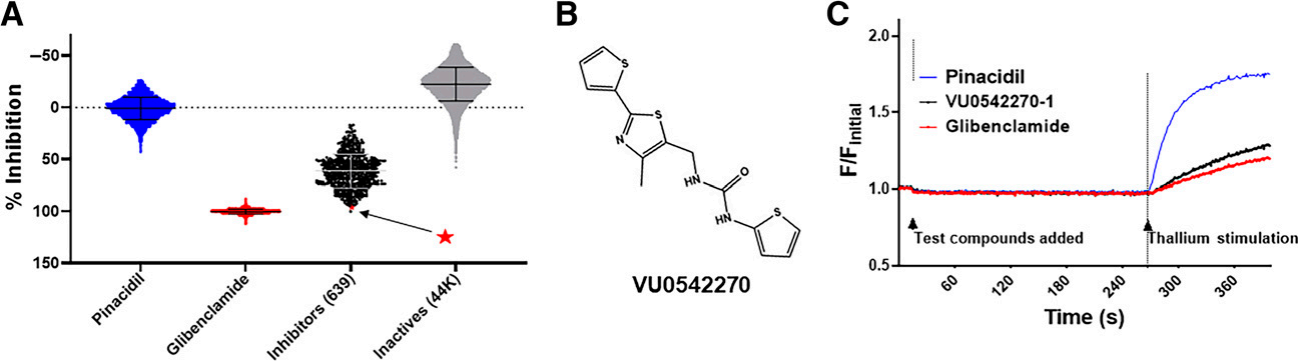
Discovery of VU0542270 in a screen of 47,872 compounds. (A) Summary of screening results. Wells were treated with 10 µM pinacidil (negative control; 0% inhibition), 10 µM pinacidil + 10 µM glibenclamide (positive control; 100% inhibition), or 10 µM pinacidil + test compounds. Each point represents the value of a single well of a 384-well plate using the slope normalized to maximal inhibition (i.e., pinacidil plus glibenclamide). Inhibitors are defined as those decreasing thallium-induced fluorescence by 3 S.D. below the mean pinacidil response and 3 mean absolute deviation below the median pinacidil plate response. Six hundred thirty-nine inhibitors (black) and 44,267 inactives are shown. Not shown are putative activators, fluorescent compounds, and retest negatives (2966). (B) Chemical structure of VU0542270. (C) Representative fluorescence traces from single wells treated with pinacidil (blue), pinacidil plus glibenclamide (red), or pinacidil plus VU0542270 (black).

We screened 47,872 compounds from the VICB library for inhibitors of pinacidil-activated Kir6.1/SUR2B channels. Screening plates contained quality control wells that received either 10 µM pinacidil alone or 10 µM pinacidil + 10 µM glibenclamide to allow calculation of Z’ for each plate. Any plates with Z’ below 0.5 were rejected from data analysis and repeated on a subsequent day. Violin plots showing screening data for pinacidil, glibenclamide, inhibitors, and inactive test compounds are shown in Fig. 2A. Percent inhibition is calculated using the slope values during thallium plus pinacidil stimulation and normalized to maximal inhibition values represented in the glibenclamide plus pinacidil controls. Test compounds that were associated with modulation of the pinacidil-stimulated response in real-time fluorescent measurements were designated as hits and selected for confirmation testing (see *Materials and Methods*). Hits were retested at 10 µM in duplicate in Kir6.1/SUR2B cells, nontransfected HEK293 cells (control cells), and Kir6.2/SUR2 cells. Of 639 compounds that retested positive in both replicate wells and did not have activity in parental cells or Kir6.2/SUR1 cells, 99 were selected for dose-response experiments based on their rank in inhibition. The most potent Kir6.1/SUR2B channel inhibitor identified from this screen was an *N*-aryl-*N*’-benzyl urea analog, which we termed VU0542270.

### VU0542270 Potency

**Fig. 3.**
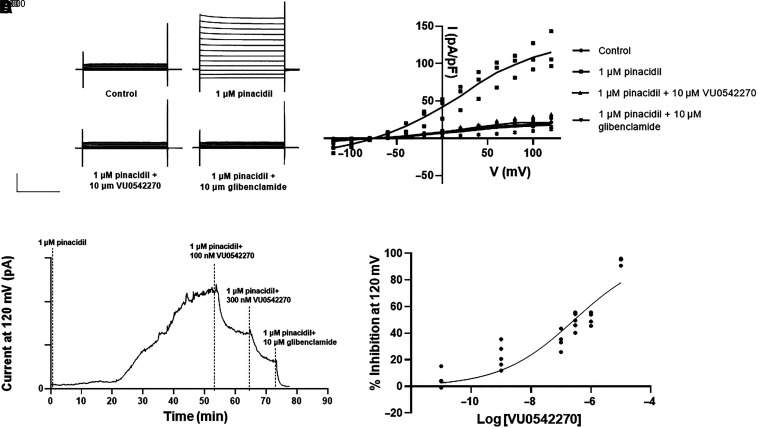
Electrophysiological characterization of VU0542270-dependent inhibition of Kir6.1/SUR2B. (A) Representative whole-cell currents recorded from HEK-293-Kir6.1/SUR2B cells bathed in control buffer (top, left), 1 µM pinacidil (top, right), 1 µM pinacidil + 10 µM VU0542270 (bottom, left), or 1 µM pinacidil + 10 µM glibenclamide (bottom, right). Cells were voltage clamped at a holding potential of −75 mV and stepped between −120 Mv and +120 mV in 20-mV increments. Current amplitude has been normalized to cell capacitance (pA/pF). (B) Current-voltage relationships measured under the indicated conditions (*n* = 4–6). (C) Representative time-course of Kir6.1/SUR2B channel activation by pinacidil (1 µM) and inhibition by the indicated dose of VU0542270 or glibenclamide (10 µM). (D) CRC data showing Kir6.1/SUR2B-dependent inhibition by VU0542270 (*n* = 4–6 at each dose). Fitting a four-parameter logistic function to the data yields an IC_50_ = 278 nM.

Voltage-clamp electrophysiology was used to confirm the inhibitory activity of VU0542270 toward Kir6.1/SUR2B. Whole-cell currents were small in control bath solution (Fig. 3A, top left panel) but increased dramatically following channel activation with 1 µM pinacidil (Fig. 3A, top right panel). Bath application of 10 µM VU0542270 in the continued presence of pinacidil led to a complete inhibition of K_ATP_ current (Fig. 3a, bottom left panel). No further inhibition was observed with the addition of 10 µM glibenclamide (Fig. 3a, bottom right panel). Mean ± S.D. current amplitude recorded at 120 mV under these conditions is summarized in Fig. 3B. A timecourse of a typical pharmacology experiment used to determine concentration-response relationships is shown in Fig. 3C. Kir6.1/SUR2B activation by 1 µM pinacidil was slow, often requiring greater than 30 minutes to reach a steady state. Once whole-cell currents stabilized in the presence of pinacidil, escalating doses of VU0542270 in the continued presence of pinacidil were applied to establish dose responses. Experiments were terminated by bath addition of 10 µM glibenclamide to estimate leak current amplitude (Fig. 3C). A fit of individual data points normalized to maximal inhibition by 10 µM VU0542270 produced an IC_50_ concentration of ∼279 nM (Fig. 3D).

### VU0542270 Is a SUR2-Specific Inhibitor

**Fig. 4.**
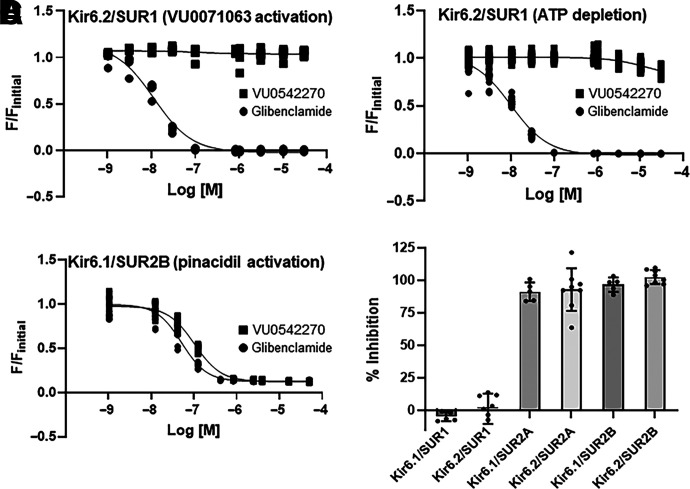
VU0542270 selectivity is mediated through SUR2. HEK-293 cells were transfected with plasmids encoding (A) Kir6.2/SUR1 (VU0071063 activation), (B) Kir6.2/SUR1 (ATP depletion), or (C) Kir6.1/SUR2B (pinacidil activation), treated with escalating doses of glibenclamide (circles) or VU0542270 (squares), and evaluated in thallium flux assays. Data are individual data (*n* = 9 wells/dose) fitted with four-parameter logistic functions to derive IC_50_ values as follows: (A) glibenclamide = 12 nM, VU0542270 = no fit, (B) glibenclamide = 10 nM, VU0542270 = no fit, (C) glibenclamide = 115 nM, and VU0542270 = 129 nM. (D) Mean ± S.D. per cent inhibition of the indicated Kir6/SUR combination with 3 µM VU0542270 (*n* = 9 wells/dose). M, molar.

VU0542270 could inhibit Kir6.1/SUR2B activity through interactions with the pore-forming Kir6.1 subunit, the regulatory SUR2B subunit, or both. As a first step toward determining VU0542270’s mechanism of action, we exploited the ability of different Kir (i.e., Kir6.1, Kir6.2) and SUR (i.e., SUR1, SUR2A, SUR2B) subunits to form functional channels when expressed together. The six possible K_ATP_ channel subunit combinations, Kir6.1/SUR1, Kir6.2/SUR1, Kir6.1/SUR2A, Kir6.2/SUR2A, Kir6.1/SUR2B, and Kir6.1/SUR2B, were reconstituted in transfected HEK-293 cells and evaluated for VU0542270 sensitivity in 10-point dose-response experiments ranging from 1 nM to 30 µM. The nonspecific K_ATP_ channel inhibitor glibenclamide was used as a positive control. Dose-response data for VU0542270 and glibenclamide against the major pancreatic and brain K_ATP_ channel subtype, Kir6.2/SUR1, activated with either VU0071063 (Raphemot et al., 2014) or metabolic inhibition (ATP depletion) are shown in Fig. 4, A and B, respectively. As expected, glibenclamide dose-dependently inhibited Kir6.2/SUR1 channels activated with VU0071063 (IC_50_ = 12.0 nM; Fig. 4A, circles) or ATP depletion (IC_50_ = 10.0 nM; Fig. 4B). Glibenclamide also inhibited pinacidil-activated Kir6.1/SUR2B (IC_50_ = 115 nM; Fig. 4C, circles). In contrast, VU0542270 failed to inhibit Kir6.2/SUR1 under either condition but inhibited Kir6.1/SUR2B dose-dependently with IC_50_ = 129.0 nM (Fig. 4C, squares). Figure 4D shows that at a dose of 3 µM, VU0542270 inhibits both Kir6.1- or Kir6.2-containing channels in complex with either SUR2A or SUR2B but not channels containing SUR1. In addition, evaluation of VU0542270 selectivity against a panel of nine other members of the Kir channel family in thallium assays revealed IC_50_ values >30 µM (Table 1). This affords a selectivity window of at least 300-fold for VU0542270-dependent inhibition of Kir6x/SUR2x over Kir6x/SUR1 and other Kir channels, making VU0542270 the most selective vascular K_ATP_ channel inhibitor reported to date.

**TABLE 1:** Selectivity profile of VU0542270 within the Kir channel family

Potassium Channel	IC_50_ (µM)
Kir6.1/SUR2B	0.11
Kir6.2/SUR1	>30
Kir1.1	>30
Kir2.1	>30
Kir2.2	>30
Kir2.3	>30
Kir3.1/Kir3.2	>30
Kir3.1/Kir3.4	>30
Kir4.1	>30
Kir4.1/Kir5.1	>30
Kir4.2	>30

### VU0542270 Structure-Activity Relationships

**Fig. 5.**
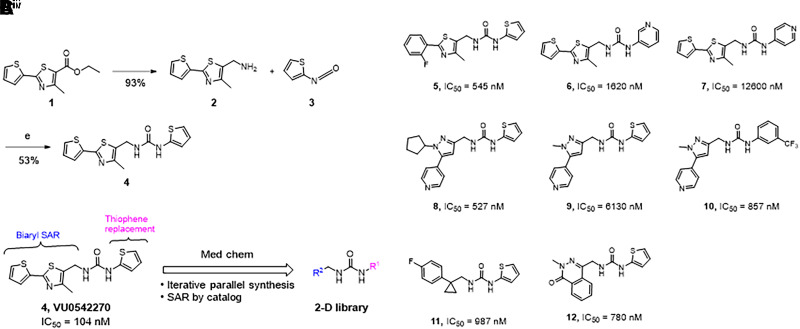
Synthesis of VU0542270 and initial SAR study plan. (A) The synthetic route for VU0542270. (Ai) LAH, THF, 0°C; (Aii) SOCl_2_, CHCl_3_, 0°C; (Aiii) NaN_3_, K_2_CO_3_, DMF, 70°C; (Aiv) PPh_3_, EtOAc, THF, 50°C. (B) Initial two-track SAR study approaches; iterative parallel synthesis and SAR by catalog. (C) Selected compounds from the initial SAR study.

As shown in Fig. 5A, the synthesis of VU0542270 was short and straightforward. The reduction, chlorination, and azide displacement followed by azide reduction sequence starting from commercial ethyl ester **1** afforded benzylamine **2** in 93% yield over four steps. Benzyl amine **2** was then reacted with isocyanate **3** under the basic condition to provide VU0542270 (**4**) in 53% yield. Considering that VU0542270 is the first reported vascular-specific K_ATP_ channel inhibitor with submicromolar potency, a medicinal chemistry approach was used to understand the chemical moieties that are required for Kir6.1/SUR2B inhibition. Forty-eight analogs of VU0542270 were synthesized and tested in thallium flux assays to establish SARs of VU0542270. For an iterative parallel synthesis approach, the left and right sides of urea were independently modified using commercially available benzylamines or isocyanates as shown in Fig. 5B. We also searched for structurally related compounds from the high-throughput screening (HTS) compound collection and conducted further investigations. Figure 5C shows a subset of compounds with SAR textures from our early SAR exploration, but detailed SAR explanations are beyond the scope of this manuscript. Briefly, the thiophene ring connected to the thiazole can be replaced by another substituted aryl ring (e.g., **5**). In general, the left side of the urea linkage tolerated more dramatic changes compared with the right side of the urea linkage (e.g., **8**, **11**, and **12** compared with **6** or **7**). Modification of the right side thiophene was rather counterproductive and resulted in very weak (e.g., **6** and **7**) or inactive compounds. More in-depth SARs will be reported in due course.

### VU0542270 Induces DA Vessel Constriction

**Fig. 6.**
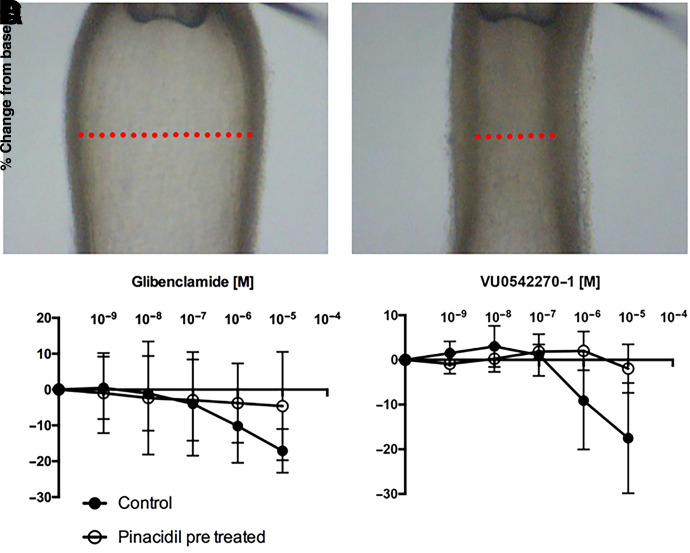
VU0542270 induces constriction of isolated mouse DA vessels. Isolated DA vessel before (A) and after (B) exposure to high extracellular potassium (i.e., 50 mM bath KCl), demonstrating vascular reactivity to membrane depolarization. (C) Vasoconstriction of isolated DA vessels in response to escalating doses of glibenclamide alone (black circles) or together with pinacidil (open circles). (D) Vasoconstriction of isolated DA vessels in response to escalating doses of VU0542270 alone (closed circles) or together with 10 µM pinacidil (open circles). Data are means ± S.D. from 4–6 vessels. M, molar.

Freshly isolated term gestation mouse DAs were challenged with increasing concentrations of glibenclamide or VU0542270 (Fig. 6). Vessels exposed to VU0542270 constricted in a concentration-dependent manner similar to that which was observed in vessels treated with glibenclamide. Vessels treated with VU0542270-1 or glibenclamide in the presence of pinacidil showed substantially blunted vasoconstrictive effects.

### DMPK Properties of VU0542270

To understand which DMPK parameters take priority in subsequent optimizations, we profiled VU0542270 in both in vitro and in vivo DMPK assays. As shown in Table 2, VU0542270 shows acceptable F_u_ values across all species in both plasma and brain (F_u,plasma_ mouse = 0.01, F_u,plasma_ rat = 0.01, F_u,plasma_ human = 0.01, F_u,brain_ mouse = 0.02, F_u,brain_ rat = 0.02). Although in vitro clearance values were rather high [*CL_int_* (mL/min per kg) = 3587 (mouse), 600 (rat), 154 (human), and predicted *CL_hep_* (mL/min per kg) = 88 (mouse), 63 (rat), 19 (human)], observed in vivo total clearance value from rat intravenous PK study was much lower (rat, CLp = 17.7 mL/min per kg). The in vitro/in vivo correlation disconnect was less pronounced when clearance was corrected by the plasma unbound fraction (CL_p,u_ = 6.03 ml/min per kg). *t*_1/2_ (human), MRT (human), and volume of steady state (L/kg) were 0.64, 0.17, and 0.20, respectively. Lastly, Kp value based on the rat intravenous PK cassette study was 0.11, indicating low brain exposure.

**TABLE 2:** Pharmacokinetic parameters of VU0542270

Property	VU0542270
Kir6.1/SUR2B IC_50_ (nM)	104
MW	335.46
cLogP	3.51
TPSA	53.5
F_u, plasma_ (mouse)	0.01
F_u, plasma_ (rat)	0.01
F_u, plasma_ (human)	0.01
F_u, brain_ (mouse)	0.02
F_u, brain_ (rat)	0.02
K_p_	0.11
*CL_int_* (mL/min per kg) (m, r, h)	3587, 600, 154
Predicted *CL_hep_* (mL/min per kg) (m, r, h)	88, 63, 19
Predicted CL_hep,u_ (mL/min per kg) (m, r, h)	27, 6.0, 1.3
Rat intravenous PK	
CL_p_ (mL/min per kg)	17.7
*t*_1/2_ (h)	0.64
MRT (h)	0.19
V_ss_ (L/kg)	0.20

CL_hep,u_, predicted hepatic clearance corrected to plasma unbound fraction; h, human; m, mouse; MW, molecular weight; r, rat; TPSA, topological polar surface area; V_ss_, volume of steady state.

## Discussion

Here, we describe the discovery and characterization of what is, to our knowledge, the first selective small-molecule inhibitor of vascular Kir6.1/SUR2B K_ATP_ channels reported to date. The most salient features of VU0542270 include its moderate potency (IC_50_ ∼100 nM), greater than 300-fold selectivity for Kir6.1/SUR2B over Kir6.2/SUR1, clean ancillary pharmacology within the Kir channel family, and ability to inhibit native vascular K_ATP_ channels expressed in DA vessels. VU0542270 therefore represents the current state of the art in vascular K_ATP_ channel inhibitors and an attractive entry point for developing novel therapeutics for disorders of vascular hypo-activity.

Many seminal advances in K_ATP_ channel pharmacology came from serendipitous discoveries or chemical optimization of existing scaffolds with medicinal chemistry. French chemist Marcel Janbon first noted in 1942 that people being treated with sulfonamides for typhoid fever exhibited symptoms of severe hypoglycemia. This was later confirmed at Auguste Loubatieres by showing that sulfonylurea drugs stimulated insulin secretion and hypoglycemia in dogs (Loubatieres-Mariani, 2007). First-generation K_ATP_ channel inhibitors were relatively weak and poorly selective; however, extensive optimization efforts over the last several decades have led to the development of numerous different structural classes of highly potent and highly selective Kir6.2/SUR1 inhibitors (Kharade et al., 2016), many of which are Food and Drug Administration (FDA)-approved for treating type 2 diabetes.

In striking contrast, the pharmacology of vascular K_ATP_ is comprised primarily of potassium channel openers, such as pinacidil, P1075, minoxidil, and levcromakalim. Levcromakalim is an FDA-approved drug that has been used in clinical trials as a “headache trigger” related to its vasodilatory effects on intracranial arteries (Ploug et al., 2012; Clement et al., 2023). Minoxidil is the active ingredient in the topical treatment Rogaine and is believed to promote hair growth by activating SUR2-containing K_ATP_ channels (Shorter et al., 2008). P1075 is a high-affinity analog of pinacidil that was shown recently in cryo-electron microscopy structures to bind between transmembrane domain (TMD)-1 and TMD2 of SUR2 to induce channel opening (Ding et al., 2022). Interestingly, the vascular-preferring K_ATP_ channel inhibitor PNU-37883A (also known as U-37883A) is an analog of P1075 that exhibits “mode switching” to an inhibitor that blocks the pore of Kir6.1 independently of SUR2 (Kovalev et al., 2004). Perhaps not surprisingly given the strong structural conservation of potassium channel pores, PNU-37883A also inhibits Kir6.2 and Kir1.1 (ROMK, *KCNJ1*) (Wang et al., 1995; Kovalev et al., 2004), making it unsuitable for use as an in vivo tool compound.

The lack of vascular-specific K_ATP_ channel inhibitors motivated us to take a molecular target-based approach to discovering novel SUR2-specific inhibitors. Several different structural classes of inhibitors that are selective for SUR2 over SUR1 and range in potencies from 100–500 nM were discovered (data not shown). We focused our initial attention on VU0542270 because of its potency and selectivity for Kir6.1/SUR2B over Kir6.2/SUR1 and other Kir channels, and several modifications to VU0542270 were made to identify key pharmacophores. Our initial scouting efforts failed to identify analogs with improved potency over VU0542270; however, they did reveal structural properties that are essential for high-affinity inhibition of Kir6.1/SUR2B. In general, modifications and/or decorations on the biaryl motif were tolerated as long as certain heteroatoms were in place. In addition, (thiophen-2-yl)urea-containing analogs tend to show better potencies than those having other heterocycles or alternative linkers. In-depth SAR trends will be reported as they become available.

An important outstanding question is: What makes VU0542270 specific for SUR2-containing K_ATP_ channels? The VU0542270 binding site and/or mechanism of action does not appear to require the distal carboxyl terminus of SUR2 since VU0542270 inhibits channels containing both SUR2A and SUR2B splice variants. We hypothesize that VU0542270 inhibits the channel through distinct interactions with residues in or near the glibenclamide binding site. Recent cryo-EM structures of Kir6.2/SUR1 and Kir6.1/SUR2B with atomic-level resolution revealed the glibenclamide binding site (Martin et al., 2017; Sung et al., 2021). In both structures, glibenclamide interacts with the same binding site in SUR1 and SUR2B. In SUR1, two arginine residues, R1246 and R1300, form the primary anchor to interact with the two oxygens of the sulfonyl group (Martin et al., 2017). In SUR2B, glibenclamide is anchored by R1213 and R1263 (Sung et al., 2021). However, glibenclamide shows higher potency against Kir6.2/SUR1 than Kir6.1/SUR2B. One key difference that may contribute to this observation is the presence of S1238 in SUR1 versus Y1205 in SUR2B. Studies have shown that SUR1-S1238Y enhances glibenclamide off rate and changes its interaction with SUR1 from irreversible to reversible as observed in SUR2B (Ashfield et al., 1999). Ongoing structural modeling and site-directed mutagenesis experiments are exploring VU0542270s molecular mechanism of action.

Gain-of-function mutations in Kir6.1 or SUR2B result in Cantu syndrome, an autosomal dominant disorder that affects multiple organ systems, including the cardiovascular system (Harakalova et al., 2012; van Bon et al., 2012; Brownstein et al., 2013; Li et al., 2013; Cooper et al., 2014) More than half of Cantu patients are born with symptomatic PDA (Nichols, 2023). We and others have shown that Kir6.1/SUR2B expression is enriched in DA vessels and may represent a druggable target for treating PDA (Shelton et al., 2014, 2018; Yarboro et al., 2018). Untreated PDA is associated with serious complications, including pulmonary edema, renal dysfunction, congestive heart failure, and bronchopulmonary dysplasia. There are currently limited pharmacotherapies available for treating PDA, leaving surgical resection as a commonly used alternative. The only two drugs approved by the FDA are indomethacin and ibuprofen, which are nonsteroidal anti-inflammatory drugs and reduce prostaglandin production by inhibiting cyclooxygenase (De Leon et al., 2023). Acetaminophen is used to lower prostaglandin production; however, its mechanism of action is unclear, and its efficacy is lower in premature infants for reasons that are unclear (Graham and Scott, 2005; El-Khuffash et al., 2014; Bardanzellu et al., 2017). Glibenclamide, when used at 100 times the clinically recommended dose, contracts and closes DA in premature mice (Nakanishi et al., 2020). This observation strongly suggests that a specific inhibitor of Kir6.1/SUR2B may offer new therapeutic opportunities for treating PDA and potentially other cardiovascular complications observed in Cantu syndrome.

Vascular K_ATP_ channels may be therapeutic targets for other diseases as well. As noted above, Levcromakalim, a Kir6.1/SUR2B channel opener, induces migraine headaches through the dilation of extracerebral arteries, suggesting that vascular K_ATP_ channel inhibitors might help treat migraines (Clement et al., 2023). K_ATP_ channels might also represent therapeutic targets for treating life-threatening vascular collapse during sepsis. Both Kir6.1 and SUR2B expression levels are upregulated in rats treated with lipopolysaccharide, a commonly used preclinical model of sepsis (Shi et al., 2010). In vivo studies have shown that glibenclamide can rapidly restore blood pressure and vasopressor responsiveness in lipopolysaccharide-treated animals while exhibiting no vascular effects on healthy control animals (Landry and Oliver, 1992; Vanelli et al., 1995; Zhang et al., 2023). A clinical trial studying whether glibenclamide can reduce vasopressor use in intensive care units failed to observe a difference between control and glibenclamide-treated groups, at least in part due to the limited dose used to minimize inhibition of pancreatic K_ATP_ and lowering of blood glucose levels (Warrillow et al., 2006). VU0542270 or optimized analogs may hold therapeutic potential for both of these common clinical problems.

In conclusion, we have discovered what is, to our knowledge, the first potent and highly selective Kir6.1/SUR2B inhibitor, VU0542270. This shows key proof of concept that a molecular target-based approach can be used successfully to identify subtype-specific inhibitors (and likely activators) of K_ATP_ channels. The discovery of VU0542270 creates new opportunities for understanding the molecular basis of subtype selectivity and mechanism of action. Furthermore, and importantly, we anticipate that VU0542270 or its optimized analogs will be useful for probing the therapeutic potential of vascular K_ATP_ channels in treating PDA, Cantu syndrome, migraine headache, and vascular collapse in sepsis without potentially confounding effects of inhibiting pancreatic and brain Kir6.2/SUR1 channels.

## Data Availability

Primary screening data not included in the article may be made available upon request.

## Abbreviations

*CL_hep_*: hepatic clearance
*CL_int_*: in vitro intrinsic clearance
CL_p_: plasma clearance
CRC: concentration-response curve
DA: ductus arteriosus
DPBS: Delbecco’s phosphate buffered saline
FDA: Food and Drug Administration
f_u_: fraction unbound
HTD: HTDialysis
K_ATP_: ATP-regulated potassium channel
Kir: inward rectifier potassium
LC-MS/MS: liquid chromatography tandem mass spectrometry
MS: mass spectrometry
PDA: patent ductus arteriosus
PK: pharmacokinetic
Q_h_: hepatic blood flow
rcf: relative centrifugal force
SAR: structure-activity-relationship
SUR: sulfonylurea receptor
*t*_1/2_: half-life
T-Rex-HEK293: tetracycline-regulated expression human embryonic kidney-293 cell
VICB: Vanderbilt Institute of Chemical Biology

## References

[B1] Aguilar-Bryan L Bryan J (1999) Molecular biology of adenosine triphosphate-sensitive potassium channels. Endocr Rev 20:101–135. 10204114 10.1210/edrv.20.2.0361

[B2] Ashfield R Gribble FM Ashcroft SJ Ashcroft FM (1999) Identification of the high-affinity tolbutamide site on the SUR1 subunit of the K(ATP) channel. Diabetes 48:1341–1347. 10342826 10.2337/diabetes.48.6.1341

[B3] Aziz Q Thomas AM Gomes J Ang R Sones WR Li Y Ng KE Gee L Tinker A (2014) The ATP-sensitive potassium channel subunit, Kir6.1, in vascular smooth muscle plays a major role in blood pressure control. Hypertension 64:523–529. 24914196 10.1161/HYPERTENSIONAHA.114.03116

[B4] Bardanzellu F Neroni P Dessì A Fanos V (2017) Paracetamol in Patent Ductus Arteriosus Treatment: Efficacious and Safe? BioMed Res Int 2017:1438038. 28828381 10.1155/2017/1438038 PMC5554551

[B5] Brownstein CA Towne MC Luquette LJ Harris DJ Marinakis NS Meinecke P Kutsche K Campeau PM Yu TW Margulies DM, (2013) Mutation of KCNJ8 in a patient with Cantú syndrome with unique vascular abnormalities - support for the role of K(ATP) channels in this condition. Eur J Med Genet 56:678–682. 24176758 10.1016/j.ejmg.2013.09.009 PMC3902017

[B6] Clement A Christensen SL Jansen-Olesen I Olesen J Guo S (2023) The ATP sensitive potassium channel (K_ATP_) is a novel target for migraine drug development. Front Mol Neurosci 16:1182515. 37456521 10.3389/fnmol.2023.1182515 PMC10338883

[B7] Cooper PE Reutter H Woelfle J Engels H Grange DK van Haaften G van Bon BW Hoischen A Nichols CG (2014) Cantú syndrome resulting from activating mutation in the KCNJ8 gene. Hum Mutat 35:809–813. 24700710 10.1002/humu.22555 PMC4277879

[B8] Cui Y Tran S Tinker A Clapp LH (2002) The molecular composition of K(ATP) channels in human pulmonary artery smooth muscle cells and their modulation by growth. Am J Respir Cell Mol Biol 26:135–143. 11751213 10.1165/ajrcmb.26.1.4622

[B9] Davis MJ Kim HJ Nichols CG (2022) K_ATP_ channels in lymphatic function. Am J Physiol Cell Physiol 323:C1018–C1035. 35785984 10.1152/ajpcell.00137.2022 PMC9550566

[B10] De Leon DD Arnoux JB Banerjee I Bergadá I Bhatti T Conwell LS Fu JF Flanagan SE Gillis D Meissner T, (2023) International Guidelines for the Diagnosis and Management of Hyperinsulinism. Horm Res Paediatr DOI: 10.1159/000531766 [published ahead of print]. 10.1159/000531766 PMC11124746 37454648

[B11] Dice JE Bhatia J (2007) Patent ductus arteriosus: an overview. J Pediatr Pharmacol Ther 12:138–146. 23055849 10.5863/1551-6776-12.3.138 PMC3462096

[B12] Ding D Wu JX Duan X Ma S Lai L Chen L (2022) Structural identification of vasodilator binding sites on the SUR2 subunit. Nat Commun 13:2675. 35562524 10.1038/s41467-022-30428-y PMC9106677

[B13] El-Khuffash A Jain A Corcoran D Shah PS Hooper CW Brown N Poole SD Shelton EL Milne GL Reese J, (2014) Efficacy of paracetamol on patent ductus arteriosus closure may be dose dependent: evidence from human and murine studies. Pediatr Res 76:238–244. 24941212 10.1038/pr.2014.82 PMC4321957

[B14] Graham GG Scott KF (2005) Mechanism of action of paracetamol. Am J Ther 12:46–55. 15662292 10.1097/00045391-200501000-00008

[B15] Gribble FM Ashfield R Ammälä C Ashcroft FM (1997) Properties of cloned ATP-sensitive K+ currents expressed in Xenopus oocytes. J Physiol 498:87–98. 9023770 10.1113/jphysiol.1997.sp021843 PMC1159236

[B16] Gribble FM Tucker SJ Seino S Ashcroft FM (1998) Tissue specificity of sulfonylureas: studies on cloned cardiac and beta-cell K(ATP) channels. Diabetes 47:1412–1418. 9726229 10.2337/diabetes.47.9.1412

[B17] Harakalova M van Harssel JJ Terhal PA van Lieshout S Duran K Renkens I Amor DJ Wilson LC Kirk EP Turner CL, (2012) Dominant missense mutations in ABCC9 cause Cantú syndrome. Nat Genet 44:793–796. 22610116 10.1038/ng.2324

[B18] Hooper CW Delaney C Streeter T Yarboro MT Poole S Brown N Slaughter JC Cotton RB Reese J Shelton EL (2016) Selective serotonin reuptake inhibitor exposure constricts the mouse ductus arteriosus in utero. Am J Physiol Heart Circ Physiol 311:H572–H581. 27371685 10.1152/ajpheart.00822.2015 PMC5142184

[B19] Inagaki N Gonoi T Clement 4th JP Namba N Inazawa J Gonzalez G Aguilar-Bryan L Seino S Bryan J (1995) Reconstitution of IKATP: an inward rectifier subunit plus the sulfonylurea receptor. Science 270:1166–1170. 7502040 10.1126/science.270.5239.1166

[B20] Inagaki N Gonoi T Clement JP Wang CZ Aguilar-Bryan L Bryan J Seino S (1996) A family of sulfonylurea receptors determines the pharmacological properties of ATP-sensitive K+ channels. Neuron 16:1011–1017. 8630239 10.1016/s0896-6273(00)80124-5

[B21] Inagaki N Gonoi T Seino S (1997) Subunit stoichiometry of the pancreatic beta-cell ATP-sensitive K+ channel. FEBS Lett 409:232–236. 9202152 10.1016/s0014-5793(97)00488-2

[B22] Kharade SV Kurata H Bender AM Blobaum AL Figueroa EE Duran A Kramer M Days E Vinson P Flores D, (2018) Discovery, Characterization, and Effects on Renal Fluid and Electrolyte Excretion of the Kir4.1 Potassium Channel Pore Blocker, VU0134992. Mol Pharmacol 94:926–937. 29895592 10.1124/mol.118.112359 PMC6041953

[B23] Kharade SV Nichols C Denton JS (2016) The shifting landscape of KATP channelopathies and the need for ‘sharper’ therapeutics. Future Med Chem 8:789–802. 27161588 10.4155/fmc-2016-0005 PMC4976861

[B24] Kharade SV Sanchez-Andres JV Fulton MG Shelton EL Blobaum AL Engers DW Hofmann CS Dadi PK Lantier L Jacobson DA, (2019) Structure-Activity Relationships, Pharmacokinetics, and Pharmacodynamics of the Kir6.2/SUR1-Specific Channel Opener VU0071063. J Pharmacol Exp Ther 370:350–359. 31201216 10.1124/jpet.119.257204 PMC6691189

[B25] Kovalev H Quayle JM Kamishima T Lodwick D (2004) Molecular analysis of the subtype-selective inhibition of cloned KATP channels by PNU-37883A. Br J Pharmacol 141:867–873. 14757705 10.1038/sj.bjp.0705670 PMC1574259

[B26] Landry DW Oliver JA (1992) The ATP-sensitive K+ channel mediates hypotension in endotoxemia and hypoxic lactic acidosis in dog. J Clin Invest 89:2071–2074. 1602014 10.1172/JCI115820 PMC295927

[B27] Li A Knutsen RH Zhang H Osei-Owusu P Moreno-Dominguez A Harter TM Uchida K Remedi MS Dietrich HH Bernal-Mizrachi C, (2013) Hypotension due to Kir6.1 gain-of-function in vascular smooth muscle. J Am Heart Assoc 2:e000365. 23974906 10.1161/JAHA.113.000365 PMC3828800

[B28] Li L Wu J Jiang C (2003) Differential expression of Kir6.1 and SUR2B mRNAs in the vasculature of various tissues in rats. J Membr Biol 196:61–69. 14724757 10.1007/s00232-003-0625-z

[B29] Lin JH Chiba M Balani SK Chen IW Kwei GY Vastag KJ Nishime JA (1996) Species differences in the pharmacokinetics and metabolism of indinavir, a potent human immunodeficiency virus protease inhibitor. Drug Metab Dispos 24:1111–1120. 8894513

[B30] Loubatières-Mariani MM (2007) [The discovery of hypoglycemic sulfonamides]. J Soc Biol 201:121–125. 17978743 10.1051/jbio:2007014

[B31] Martin GM Yoshioka C Rex EA Fay JF Xie Q Whorton MR Chen JZ Shyng SL (2017) Cryo-EM structure of the ATP-sensitive potassium channel illuminates mechanisms of assembly and gating. eLife 6:e24149. 28092267 10.7554/eLife.24149 PMC5344670

[B32] McClenaghan C Nichols CG (2022) Kir6.1 and SUR2B in Cantú syndrome. Am J Physiol Cell Physiol 323:C920–C935. 35876283 10.1152/ajpcell.00154.2022 PMC9467476

[B33] McClenahan SJ Kent CN Kharade SV Isaeva E Williams JC Han C Terker A Gresham 3rd R Lazarenko RM Days EL, (2022) VU6036720: The First Potent and Selective In Vitro Inhibitor of Heteromeric Kir4.1/5.1 Inward Rectifier Potassium Channels. Mol Pharmacol 101:357–370. 35246480 10.1124/molpharm.121.000464 PMC9092466

[B34] Nakanishi T Baldwin HS Fineman JR Yamagishi H (2020) Molecular Mechanism of Congenital Heart Disease and Pulmonary Hypertension, in *SpringerLink and Takao International Symposium on Molecular Mechanism of Cardiopulmonary D*; 2020; Springer, Singapore.

[B35] Nichols CG (2006) KATP channels as molecular sensors of cellular metabolism. Nature 440:470–476. 16554807 10.1038/nature04711

[B36] Nichols CG (2023) Personalized Therapeutics for K_ATP_-Dependent Pathologies. Annu Rev Pharmacol Toxicol 63:541–563. 36170658 10.1146/annurev-pharmtox-051921-123023 PMC9868118

[B37] Nichols CG Singh GK Grange DK (2013) KATP channels and cardiovascular disease: suddenly a syndrome. Circ Res 112:1059–1072. 23538276 10.1161/CIRCRESAHA.112.300514 PMC3660033

[B38] Ploug KB Amrutkar DV Baun M Ramachandran R Iversen A Lund TM Gupta S Hay-Schmidt A Olesen J Jansen-Olesen I (2012) K(ATP) channel openers in the trigeminovascular system. Cephalalgia 32:55–65. 22144717 10.1177/0333102411430266

[B39] Quayle JM Nelson MT Standen NB (1997) ATP-sensitive and inwardly rectifying potassium channels in smooth muscle. Physiol Rev 77:1165–1232. 9354814 10.1152/physrev.1997.77.4.1165

[B40] Raphemot R Swale DR Dadi PK Jacobson DA Cooper P Wojtovich AP Banerjee S Nichols CG Denton JS (2014) Direct activation of β-cell KATP channels with a novel xanthine derivative. Mol Pharmacol 85:858–865. 24646456 10.1124/mol.114.091884 PMC4014665

[B41] Shelton EL Ector G Galindo CL Hooper CW Brown N Wilkerson I Pfaltzgraff ER Paria BC Cotton RB Stoller JZ, (2014) Transcriptional profiling reveals ductus arteriosus-specific genes that regulate vascular tone. Physiol Genomics 46:457–466. 24790087 10.1152/physiolgenomics.00171.2013 PMC4080279

[B42] Shelton EL Singh GK Nichols CG (2018) Novel drug targets for ductus arteriosus manipulation: Looking beyond prostaglandins. Semin Perinatol 42:221–227. 29880312 10.1053/j.semperi.2018.05.004 PMC6064654

[B43] Shi W Cui N Wu Z Yang Y Zhang S Gai H Zhu D Jiang C (2010) Lipopolysaccharides up-regulate Kir6.1/SUR2B channel expression and enhance vascular KATP channel activity via NF-kappaB-dependent signaling. J Biol Chem 285:3021–3029. 19959479 10.1074/jbc.M109.058313 PMC2823456

[B44] Shorter K Farjo NP Picksley SM Randall VA (2008) Human hair follicles contain two forms of ATP-sensitive potassium channels, only one of which is sensitive to minoxidil. FASEB J 22:1725–1736. 18258787 10.1096/fj.07-099424

[B45] Shyng S Nichols CG (1997) Octameric stoichiometry of the KATP channel complex. J Gen Physiol 110:655–664. 9382894 10.1085/jgp.110.6.655 PMC2229396

[B46] Sung MW Yang Z Driggers CM Patton BL Mostofian B Russo JD Zuckerman DM Shyng SL (2021) Vascular K_ATP_ channel structural dynamics reveal regulatory mechanism by Mg-nucleotides. Proc Natl Acad Sci USA 118:e2109441118. 34711681 10.1073/pnas.2109441118 PMC8694068

[B47] van Bon BW Gilissen C Grange DK Hennekam RC Kayserili H Engels H Reutter H Ostergaard JR Morava E Tsiakas K, (2012) Cantú syndrome is caused by mutations in ABCC9. Am J Hum Genet 90:1094–1101. 22608503 10.1016/j.ajhg.2012.04.014 PMC3370286

[B48] Vanelli G Hussain SN Aguggini G (1995) Glibenclamide, a blocker of ATP-sensitive potassium channels, reverses endotoxin-induced hypotension in pig. Exp Physiol 80:167–170. 7734136 10.1113/expphysiol.1995.sp003832

[B49] Wang T Wang WH Klein-Robbenhaar G Giebisch G (1995) Effects of a novel KATP channel blocker on renal tubule function and K channel activity. J Pharmacol Exp Ther 273:1382–1389. 7791111

[B50] Warrillow S Egi M Bellomo R (2006) Randomized, double-blind, placebo-controlled crossover pilot study of a potassium channel blocker in patients with septic shock. Crit Care Med 34:980–985. 16484892 10.1097/01.CCM.0000206114.19707.7C

[B51] Yamada M Isomoto S Matsumoto S Kondo C Shindo T Horio Y Kurachi Y (1997) Sulphonylurea receptor 2B and Kir6.1 form a sulphonylurea-sensitive but ATP-insensitive K+ channel. J Physiol 499:715–720. 9130167 10.1113/jphysiol.1997.sp021963 PMC1159289

[B52] Yarboro MT Durbin MD Herington JL Shelton EL Zhang T Ebby CG Stoller JZ Clyman RI Reese J (2018) Transcriptional profiling of the ductus arteriosus: Comparison of rodent microarrays and human RNA sequencing. Semin Perinatol 42:212–220. 29910032 10.1053/j.semperi.2018.05.003 PMC6064668

[B53] Zhang DD Duan XP Mutig K Rausch F Xiao Y Zheng JY Lin DH Wang WH (2023) Calcineurin inhibitors stimulate Kir4.1/Kir5.1 of the distal convoluted tubule to increase NaCl cotransporter. JCI Insight 8:e165987. 36821372 10.1172/jci.insight.165987 PMC10132170

